# 595. Embrace Your Role: A Structured Preceptorship Model to Enhance Burn Nurse Orientation and Preceptor Effectiveness

**DOI:** 10.1093/jbcr/irag033.389

**Published:** 2026-04-06

**Authors:** Josie Hickerson, Becca Anderson, Steven A Kahn

**Affiliations:** South Carolina Burn Center at Medical University of South Carolina, Charleston, SC; South Carolina Burn Center, Charleston, SC; Medical University of South Carolina, Charleston, SC

## Abstract

**Introduction:**

Effective orientation is crucial in a specialized burn unit. In the setting of increased nursing turnover across the US, the authors hypothesized that implementing standardized tools for preceptors would improve the experience for both preceptors and orientees by increasing readiness, confidence, satisfaction, and reduce anxiety.

**Methods:**

Two structured orientation pathways were developed based on core CBRN competencies recommended by the American Burn Association (ABA): a novice nurse pathway (12 weeks) and an experienced nurse new to burn care pathway (8 weeks). Benner’s Novice to Expert principles were used to develop a model based on biweekly evaluations to guide structured feedback, outline burn skills, objectives, and set goals. Additional tools to aid in hand off and bridge communication included observation logs, multidisciplinary shadow week reflection, and burn dressing guides.

A mixed-method approach was used to collect data through anonymous surveys using Likert scales and short answer questions, then analyzed by Wilcoxon Signed – Rank test and a Wilcoxon Rank - Sum test accordingly.

**Results:**

Subjective responses from orientees conveyed a key barrier to learning stemmed from switching between numerous preceptors, feeling they had to start over each time. Preceptors in contrast expressed their biggest challenge consisted in tracking an orientee’s progress without structure, only memory. After implementation of the new pathways, survey results demonstrated statistically significant increases in preceptors’ understanding of the evaluation process, ability to regularly and consistently re-evaluate the orientee, and their understanding of the orientee’s strengths and weaknesses (Table 1.)

For orientees, preceptor variability was reduced to maintain exposure to varied clinical approaches without compromising training. Small improvements were noted across responses, with perception of support remaining high (10 (7-10) to 10 (10-10)); however, the sample size limited statistical analysis for significance, (Table 2). Two negative outliers were noted in control responses (anxiety 10/10, satisfaction 1/10) but were absent after implementation.

**Conclusions:**

A structured preceptor program significantly improves the orientation experience, especially on clarity and consistency of evaluations. Improving the preceptor experience showed promising downstream trends in improving the orientees’ preparedness, burn competency, satisfaction, and anxiety, though sample size limited statistical confirmation. Findings supported structured tools as a foundation for orientation improvement, with adjustments to address gaps.

**Applicability of Research to Practice:**

Quality improvement project to improve the education of new nurses. To create a more confident staff and increase burn competence, thus ensuring quality of care for burn patients.

**Funding for the study:**

N/A.

